# Integration analysis of *cis*- and *trans*-regulatory long non-coding RNAs associated with immune-related pathways in non-small cell lung cancer

**DOI:** 10.1016/j.bbrep.2024.101832

**Published:** 2024-10-28

**Authors:** Yinqiang Liu, Hongjv Yang, Guoli Lv, Jin Duan, Wei Zhao, Yunfei Shi, Youming Lei

**Affiliations:** Department of Geriatric Thoracic Surgery, the First Hospital of Kunming Medical University, Kunming, China

**Keywords:** Non-small cell lung cancer, Long non-coding RNAs, *cis-*Regulation, *trans*-Regulation, Tumorigenesis

## Abstract

**Background:**

Long non-coding RNAs (lncRNAs) are importantly involved in the initiation and progression of non-small cell lung cancer (NSCLC). However, the classification and mechanisms of lncRNAs remain largely elusive.

**Aim:**

Hence, we addressed this through bioinformatics analysis.

**Methods and results:**

We utilized microarray technology to analyze lncRNAs and mRNAs in twenty paired NSCLC tumor tissues and adjacent normal tissues. Gene set enrichment analysis, Kyoto Encyclopedia of Genes and Genomes, and Gene Ontology were conducted to discern the biological functions of identified differentially expressed transcripts. Additionally, networks of lncRNA-mRNA co-expression, including *cis*-regulation, lncRNA-transcription factor (TF)-mRNA, *trans*-regulation, and lncRNA-miRNA-mRNA interactions were explored. Furthermore, the study examined differentially expressed transcripts and their prognostic values in a large RNA-seq dataset of 1016 NSCLC tumors and normal tissues extracted from the Cancer Genome Atlas (TCGA). The analysis revealed 391 lncRNAs and 344 mRNAs with differential expression in NSCLC tumor tissues compared to adjacent normal tissues. Subsequently, 43,557 co-expressed lncRNA-mRNA pairs were identified, including 27 lncRNA-mRNA pairs in cis, 9 lncRNA-TF-mRNA networks, 34 lncRNA-mRNA pairs in trans, and 8701 lncRNA-miRNA-mRNA competing endogenous RNA (ceRNA) networks. Notably, these lncRNAs were found to be involved in immune-related pathways. Six significant transcripts, including NTF4, PTPRD-AS, ITGA11, HID1-AS1, RASGRF2-AS1, and TBX2-AS1, were identified within the ceRNA network and *trans*-regulation.

**Conclusion:**

This study brings important insights into the regulatory roles of lncRNAs in NSCLC, providing a fresh perspective on lncRNA research in tumor biology.

## Highlights

• Genome-wide profiling of long non-coding RNAs (lncRNAs) in NSCLC datasets.
•*Cis*- and *trans*-lncRNAs were identified in NSCLC.
•Both *cis*- and *trans*-lncRNAs regulate key immune-related pathways in NSCLC.

## 1 Introduction

Lung cancer is one of the most prevalent cancer types and is a leading cause of cancer-related mortality [1]. In 2022, lung cancer leads to approximately 870,982 new cases and 505,618 deaths in China [2]. Over 80% of lung cancer have been diagnosed as non-small cell lung cancer (NSCLC), representing a significant and serious subtype of the disease [3]. Multiple treatment strategies have been developed for cancer management, aimed at enhancing the five-year survival (OS) rates of patients with non-small cell lung cancer (NSCLC) over the past decades [[4], [5], [6]]. Despite advancements in treatment strategies, the five-year overall survival (OS) rate for non-small cell lung cancer (NSCLC) is still low, ranging from 10% to 20% according to the Global Surveillance of Trends in Cancer Survival 2000–2014 (CONCORD-3) [7]. These findings underscore the need to explore additional diagnostic and therapeutic biomarkers for NSCLC, thereby significantly enhancing the clinical outcomes for patients with this disease.

In recent years, numerous studies highlighted the pivotal roles of lncRNAs in cancer, demonstrating their regulation of tumor initiation and progression, and their correlation with the prognosis and survival outcomes of patients [[8], [9], [10]]. Especially, lncRNA pro-transition associated RNA (PTAR) can be an oncogene in NSCLC by increasing cell proliferation, migration, and invasion [11]. While lncRNAs have been implicated in various oncogenic processes, fetal-lethal non-coding developmental regulatory RNA (FENDRR) acts as a tumor suppressor in NSCLC, inhibiting tumor cell growth, migration, and invasion [12]. Although numerous lncRNAs exert vital roles in NSCLC, the focus regarding attention on the mechanism of action is their RNA products in the past years. Recent research indicates that not only the products of RNA species exert important roles, but also the lncRNA locus separately exerts regulatory functions [13,14].

About 10%–20% of human transcripts are identified as protein-coding RNAs, and 80%–90% of transcripts are non-protein-coding RNAs (non-coding RNAs) [15]. LncRNAs, a subclass of non-coding RNAs characterized by transcripts longer than 200 nucleotides, do not encode proteins but possess extensive regulatory capabilities, influencing a broad spectrum of cellular processes [16,17]. LncRNAs are heterogeneous molecules in various cancers due to their diverse locus and function [18]. LncRNAs are broadly classified into different categories according to relative genomic position to the protein-coding RNAs, including sense, antisense, intronic, intergenic, and bidirectional [16,19,20]. Indeed, lncRNAs play multifaceted roles in both *cis* and *trans* regulation, organization of nuclear, and gene modulation, sometimes even encoding small proteins themselves [[21], [22], [23]]. It has been challenging to understand the biological function of several lncRNAs in regulating biological and pathological processes mediated by the lncRNAs themselves. However, to date, our understanding of the expression and regulatory mechanisms of lncRNAs in NSCLC remains limited. In recent years, advancements in next-generation sequencing (NGS) have enabled the confirmation of numerous biomarkers and underlying mechanisms in lung cancer through bioinformatics analysis [[24], [25], [26], [27]].

This study aims to identify the landscape of lncRNA expression and explore the function of lncRNAs in NSCLC using bioinformatics analysis. We identified lncRNAs and protein-coding transcripts that showed differential expressions based on microarray analysis. The *cis*- and *trans*-regulatory lncRNAs and their regulated biological pathways were then explored in NSCLC. Importantly, TBX2-AS1, NTF4, PTPRD-AS, ITGA11, RASGRF2-AS1, and HID1-AS1 were identified as potential therapeutic biomarkers for NSCLC.

## 2 Material and methods

### 2.1 Specimen

Three tumor samples and normal tissues were collected from patients diagnosed with NSCLC. The cohort consisted of two females and one male, with a median age of 64 years (50–70). All patients have signed informed consent for their inclusion in this study. Importantly, samples were procured before any pre-operative radiation or chemotherapy treatments. After the pathological examination by three pathologists, the samples were saved in liquid nitrogen. Our research has been supported by the Ethics Committee of the First Hospital of Kunming Medical University.

### 2.2 RNA extraction, cDNA library preparation, and microarray analysis

Total RNA was isolated from fresh NSCLC tumor tissues and normal tissues using TRzol reagent (Thermo Fisher Scientific, Waltham, MA, USA) following the manufacturer's protocol. The concentration and quality of RNA were assessed using the NanoDrop ND-2000 spectrophotometer (Thermo Fisher Scientific, Waltham, MA, USA), and the integrity of RNA was evaluated using an Agilent Bioanalyzer 2100 (Agilent Technologies, Santa Clara, CA, USA) according to the manufacturer's instructions. Subsequently, purified single-strand RNA was reverse transcribed to generate double-strand cDNA and labeled with Cyanine 3-CTP (Cy3). The Cy3-labeled cDNA was then hybridized onto the Agilent Human ceRNA Microarray 2019 (4∗180k, Design ID:086188). Finally, the hybridized slides were scanned using the G2505C Agilent Scanner (Agilent Technologies).

### 2.3 Quality control and data standardization

After screening the original images of slides, the raw data was extracted using the Feature Extraction software (version 10.7.1.1, Agilent Technologies). Subsequently, the raw data were normalized and analyzed using the R software package. The coefficient of variable (CV) value of each sample was used to the stability of the signal detected by the chip repeat probe. The correlation between samples was used to measure the correlation between these samples. Principal component analysis (PCA) was used to reveal the distribution of the main information of each sample.

### 2.4 Differential analysis for lncRNAs and mRNAs

LncRNAs and mRNAs with different expressions were identified using the student's t-test [28], the threshold adjusted as log2(Fold change, FC)≥2 and *P* ≤ 0.05. The volcano plots and heatmaps were drawn to show the significantly differential expressed lncRNAs and mRNAs.

### 2.5 *Biofunction analysis of DE-mRNA*s

Gene Ontology (GO) analysis [29,30], encompassing biological process (BP), cellular component (CC), and molecular function (MF), was conducted using bioinformatics tools available at OECloud (https://cloud.oebiotech.cn). The significance of each GO term was assessed utilizing the Hypergeometric distribution algorithm, as previously described [31]. KEGG pathway enrichment analysis was carried out using the Hypergeometric distribution algorithm based on the KEGG database [32].

### 2.6 C*orrelation analysis for co-expression lncRNA-mRNA pairs*

The correlation between lncRNAs and mRNAs with differential expressions (DE-mRNAs) was evaluated using the Pearson correlation analysis. LncRNA-mRNA pairs were revealed based on a correlation coefficient greater than 0.8 and *P* < 0.05. The lncRNA-mRNA pairs were visualized using the Circos tool [32].

### 2.7 *Cis*- and *trans*-regulation analysis

Furthermore, we explored the regulatory manners of lncRNAs according to the distances between the protein-coding transcripts and lncRNAs. First, we investigated the potential *cis*-regulation of lncRNAs on their neighboring protein-coding transcripts, focusing on those located within a 100 kb distance from the lncRNA locus. The neighboring protein-coding transcripts of lncRNAs were screened using the FEELnc tool [33], and the *cis*-regulatory lncRNA-mRNA pairs were identified by overlapping with the above lncRNA-mRNA pairs. Second, we explored the *trans*-regulatory lncRNAs on protein-coding transcripts using RIsearch-2.0 according to the filter criteria, the direct binding sites of lncRNAs sequences on protein-coding transcript sequences more than 10 bases, and the binding free emery less than 100 [34]. The *trans*-regulatory lncRNA-mRNA pairs were screened by overlapping with the above lncRNA-mRNA pairs and visualized using a network tool [35]. Third, lncRNAs recruited TFs to the specific DNA sequence region (such as promoters) to initiate transcriptional activity, or multiple TFs bind to a lncRNA to regulate molecular mechanisms. We analyzed transcription factor (TF) binding motifs within the regulatory regions of lncRNAs, specifically within a range of ± 1 kb from the lncRNA start sites. Potential TFs binding to lncRNAs were predicted using JASPAR [36]. We constructed the lncRNA-TF-mRNA ternary regulatory network based on data from the Gene Transcription Regulation Database (GTRD) [37] and the above lncRNA-mRNA pairs, and the network was visualized network tool [35].

### 2.8 Pathway and network enrichment analysis

The protein-coding transcripts of the *cis*-, *trans*-regulatory lncRNA-mRNA pairs, and ceRNA network were annotated and enriched via the clusterProfiler package.

### 2.9 TCGA data analysis

The RNA-seq dataset was obtained from 1016 NSCLC tumors and normal tissues from the Cancer Genome Atlas (TCGA) database, accessible at https://www.cancer.gov/about-nci/organization/ccg/research/structural-genomics/tcga. This dataset includes 515 samples of TCGA-LUAD (lung adenocarcinoma) and 501 samples of TCGA-LUSC (lung squamous cell carcinoma). The relationship between significant transcripts and clinical phenotypes, including age, gender, stages, TNM, lymph node count information, and the relationship between expression of the transcripts and survival were investigated.

## 3 Results

### 3.1 Identification of the DE-lncRNAs and DE-mRNAs in NSCLC

**Fig. 1:**
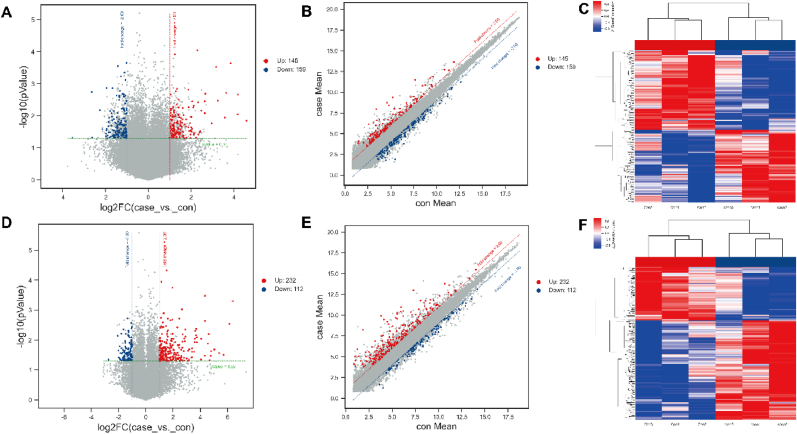
Identification of the DE-lncRNAs and DE-mRNAs in NSCLC. **(A).** The volcano plots illustrated the different distributions of the DE-lncRNAs. Red points represented upregulated lncRNAs. Point represented downregulated lncRNAs. **(B).** The scatter plots exhibited the expression levels of DE-lncRNAs between NSCLC tumor and adjacent normal tissues. Red points represented upregulated lncRNAs. Point represented downregulated lncRNAs. **(C).** Heatmap of the unsupervised hierarchical clustering showing the distinction of DE-lncRNAs between NSCLC tumor and adjacent normal tissues. **(D).** The volcano plots exhibited the distribution of DE-mRNAs. Red points represented upregulated mRNAs. Point represented downregulated mRNAs. **(E).** The scatter plots exhibited the expression levels of DE-mRNAs between NSCLC tumor and adjacent normal tissues. Red points represented upregulated mRNAs. Point represented downregulated mRNAs. **(F).** Heatmap of the unsupervised hierarchical clustering showing the distinction of DE-mRNAs between NSCLC tumor and adjacent normal tissues. (For interpretation of the references to color in this figure legend, the reader is referred to the Web version of this article.)

In this study, a Microarray analysis was carried out to investigate the transcriptome alteration in NSLCLC. After quality control and data standardization (Fig. S1), 391 lncRNAs were identified, which were preferably distributed in Chr1, Chr2, Chr6, Chr10, Chr11, and Chr17 (Fig. S2A). More than 70 % of lncRNAs are longer than 500 bp (Fig. S2B). We found that the highest percentage of lncRNAs located in 1–3 exons (Fig. S2C). The types of lncRNAs were identified as exon type (27.55 %), intron type (23.14 %), and intergenic region type (49.31 %) (Figs. S2D–E). Furthermore, 304 DE-lncRNAs (145 upregulated and 159 downregulated) were identified [log2(FC) > 2 and *P* < 0.05] (Fig. 1A–C, Table S1). Similarly, a total of 344 DE-mRNAs (232 upregulated and 112 downregulated) were identified [log2(FC) > 2 and *P* < 0.05] (Fig. 1D–F, Table S2) in NSCLC tumor tissues compared to that in normal tissues.

### 3.2 Enrichment analysis of mRNAs with differential expressions

**Fig. 2:**
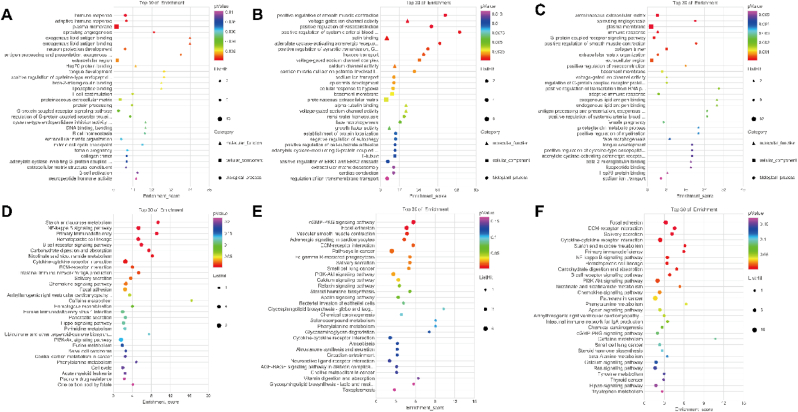
Bubble plots of GO and KEGG pathway enrichment. **(A).** Top 30 GO annotations of upregulated DE-mRNAs in NSCLC, including MF, CC, and BP terms. **(B).** Top 30 GO annotations of downregulated DE-mRNAs in NSCLC, including MF, CC, and BP terms. **(C).** Top 30 GO annotations of DE-mRNAs in NSCLC, including MF, CC, and BP terms. **(D).** Top 30 KEGG pathway enrichment analysis of upregulated DE-mRNAs in NSCLC. **(E).** Top 30 KEGG pathway enrichment analysis of downregulated DE-mRNAs in NSCLC. **(F).** Top 30 KEGG pathway enrichment analysis of DE-mRNAs in NSCLC.

We next explored the function of these DE-mRNAs. The results of GO analysis indicated that 344 mRNAs were enriched in 111 GO terms, including 70 biological processes, 19 cellular components, and 22 molecular functions (Table S3). As shown in Fig. 2A and S3A, the biological processes mainly included G-protein coupled receptors, sprouting angiogenesis, immune response, and extracellular matrix organization. The cellular components were linked with the plasma membrane, collagen trimer, extracellular region, and basement membrane. The molecular functions were involved in voltage-gated ion channel activity, endogenous lipid antigen binding, beta-2-microglobulin binding, and lipopeptide binding.

Respectively, GO analysis showed that the upregulated mRNAs are involved in multiple biological pathways and cellular components, such as the innate and adaptive immune response, plasma membrane, collagen trimer, Hsp70 protein binding, and beta-2-microglobulin binding (Fig. 2B and S3B, Table S4). In contrast, the downregulated mRNAs were found to be associated with several cellular components and functions, including positive regulation of smooth muscle contraction, vasoconstriction, systemic arterial blood pressure, proteinaceous extracellular matrix, T-tubule, actin binding, and calcium channel activity (Fig. 2C and S3C, Table S5).

Moreover, the KEGG pathway enrichment analysis revealed that differentially expressed mRNAs were enriched in 17 pathways (Table S6). Of these, upregulated mRNAs were enriched in 13 pathways, such as starch and sucrose metabolism, NF-кB signaling pathway, primary immunodeficiency, and carbohydrate digestion (Fig. 2E, Table S7). The downregulated mRNAs enriched in 11 pathways, such as focal adhesion, receptor-ECM interaction, and pathways in cancer (Fig. 2F, Table S8). These findings suggest that these enriched pathways are involved in biological processes related to immune response.

### 3.3 Comprehensive analysis of correlation between lncRNAs and mRNAs in NSCLC

**Fig. 3:**
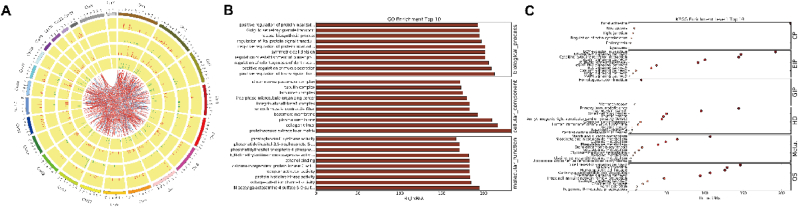
Comprehensive analysis of the correlation between DE-lncRNAs and DE-mRNAs in NSCLC **(A).** Circos map of lncRNA-mRNA pairs with criteria Correlation Coefficient>0.08 and P < 0.05. outermost layer illustrated the distribution of 23 pairs of chromosomes; The second and third layers displayed the distribution of DE-mRNAs on chromosomes, red indicates upregulated mRNAs, green indicates downregulated mRNAs; The fourth and fifth layers illustrated the distribution of DE-lncRNAs on chromosomes, es, red indicates upregulated mRNAs, green indicates downregulated mRNAs; Internal connection wires indicates top 500 lncRNA-mRNA pairs in circus map. **(B).** The bar plots revealed GO enrichment, including the top 10 Molecular Function (MF), top 10 Cellular Component (CC), and top 10 Biological Processes (BP). **(C).** Bubble plots illustrated KEGG pathway enrichment, including Cellular Processes (CP), Environmental Information Processing (EIP), Genetic Information Processing (GIP), Human Diseases (HD), Metabolism (Meta.), Organismal Systems (OS). (For interpretation of the references to color in this figure legend, the reader is referred to the Web version of this article.)

Next, we investigated the correlation between DE-lncRNAs and DE-mRNAs using Pearson correlation analysis. In total, 43,557 lncRNA-mRNA pairs were screened (Correlation Coefficient ≥0.8 and *P* ≤ 0.05) (Table S9). The distribution of these lncRNA-mRNA pairs in chromosomes is illustrated in Fig. 3A, and these lncRNA-mRNA pairs were observed with no chromosome distribution preference. We further explored the DE-mRNA that co-expressed with lncRNAsand found that pathways associated with various biological processes were enriched, such as the cellular response to chemical stimulus and mucus secretion. These are also involved in cellular components including collagen trimer, and basement membrane. The molecular functions of these genes are involved in voltage-gated ion channel activity, and protein kinase activities (Fig. 3B, Tables S10–S11). Additionally, KEGG pathway enrichment results indicated that they were associated with six modules. These modules contained pathways such as necroptosis, tight junction, receptor-ECM interaction, apelin signaling, PI3K-AKT signaling, homologous recombination, primary immunodeficiency, pathways in cancer, as well as starch and sucrose metabolism (Fig. 3C, Tables S12–13).

### 3.4 Identification of cis-regulatory LncRNAs and enrichment analysis of their correlative transcripts

**Fig. 4:**
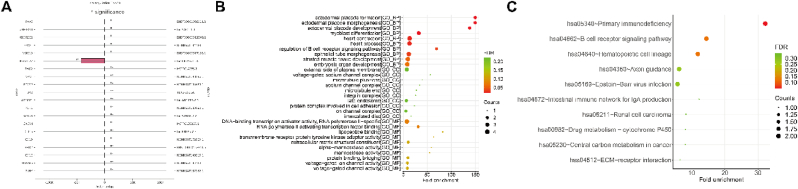
Identification of *cis*-regulatory lncRNAs and enrichment analysis of their correlative transcripts **(A).** The bar plots of the *cis*-regulatory lncRNA-mRNA pairs. The X-axis indicates the distances between lncRNA and mRNA, (+) indicates downstream of lncRNA, (−) indicates upstream of lncRNA; the Left of the Y-axis indicates mRNA, and the right of the Y-axis indicates lncRNAs. **(B).** The bubble plots of GO enrichment, including top 10 BP, top 10 CC, and top 10 MF terms. **(C).** The bubble plots of top 10 KEGG enrichment.

To explore the *cis*-regulatory effects of lncRNAs on protein-coding genes, we analyzed those transcripts located within a 100 kb distance from the lncRNA loci. The results showed that 27 *cis*-regulatory lncRNAs and their regulated protein-coding transcripts were identified from above 43,557 lncRNA-mRNA pairs (Table S14). In Fig. 4A, the top 20 *cis*-regulatory lncRNAs and protein-coding genes regulated by them are depicted, along with the distances between the lncRNAs and protein-coding transcripts. Moreover, the functional enrichment analysis suggested that *cis*-regulatory lncRNA-mRNA pairs were mainly associated with myoblast differentiation, heart contraction, heart process, and epithelial tube morphogenesis (Fig. 4B, Table S15). Moreover, KEGG pathway enrichment analysis suggested that the *cis*-regulatory lncRNA-mRNA pairs enriched in primary immunodeficiency (Fig. 4C, Table S16).

Identification of the Trans-Regulatory LncRNAs and Enrichment Analysis of Their Correlative Transcripts.

**Fig. 5:**
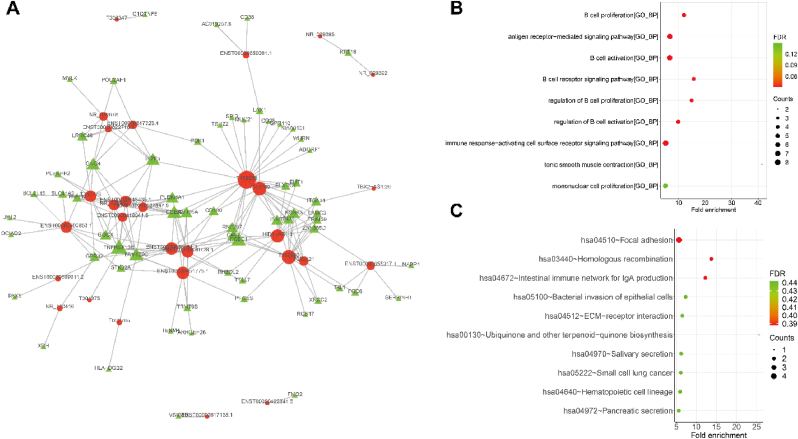
Identification of the *trans*-regulatory lncRNAs and enrichment analysis of their correlative transcripts **(A).** The binary network of the top 500 *trans*-regulatory lncRNA-mRNA pairs, red indicates lncRNAs, green indicates mRNAs, and the size of the node indicates counts of genes. **(B).** The bubble plots of GO enrichment, including top 10 BP terms. **(C).** The bubble plots of top 10 KEGG enrichment. (For interpretation of the references to color in this figure legend, the reader is referred to the Web version of this article.)

Next, we explored the *trans*-regulatory lncRNAs on protein-coding transcripts, and the results showed that 2078 *trans*-regulatory lncRNA-mRNA pairs were identified from above 43,557 lncRNA-mRNA pairs and that 34 lncRNAs were involved in *trans*-regulation (Table S17). The *trans*-regulatory lncRNA-mRNA pairs are depicted in Fig. 5A. The results of the GO enrichment analysis revealed that *trans*-regulatory lncRNA-mRNA pairs are primarily enriched in various immune-related biological processes, including B cell proliferation, antigen receptor-mediated signaling pathway, and B cell activation (Fig. 5B, Table S18). KEGG pathway enrichment analysis indicated that *trans*-regulatory lncRNA-mRNA pairs mainly enriched in multiple pathways, including focal adhesion, homologous recombination, bacterial invasion of epithelial cells, ECM-receptor interaction, ubiquinone, and another terpenoid-quinone biosynthesis, salivary secretion, small cell lung cancer, Hematopoietic cell lineage, and pancreatic secretion (Fig. 5C, Table S19).

### 3.5 Identification of LncRNA-TF pairs

**Fig. 6:**
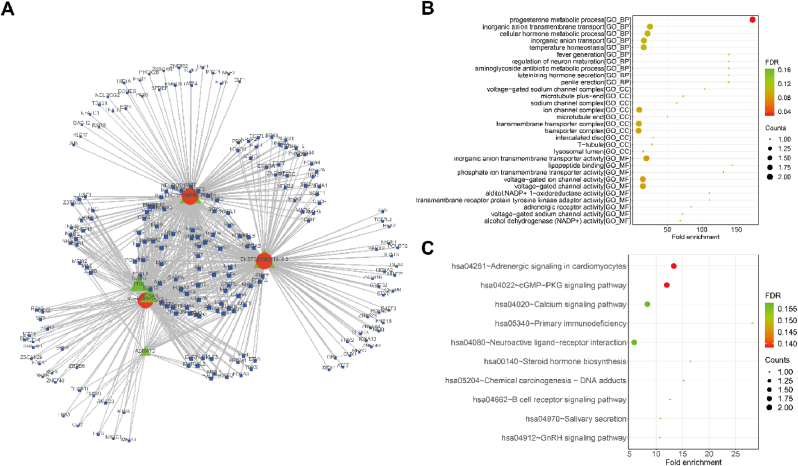
Identification of lncRNA-TF pairs **(A).** The ternary network of top 500 lncRNA-TF-mRNA with degree>2, red indicates lncRNAs, green indicates mRNAs, blue indicates TF, and the size of the node counts of genes. **(B).** The bubble plots of GO enrichment, including top 10 BP, top 10 CC, and top 10 MF terms.**(C).** The bubble plots of top 10 KEGG enrichment. (For interpretation of the references to color in this figure legend, the reader is referred to the Web version of this article.)

To investigate the potential roles of transcription factors (TFs) on lncRNAs, we identified TF binding sites in the regulatory regions on these lncRNAs, spanning 1 kb upstream and downstream of the lncRNA transcriptional start sites. The lncRNA-TF pairs were identified from the previously established 27 *cis*-regulatory lncRNA-mRNA pairs. A lncRNA-TF-mRNA network was built using data from JASPAR and GTRD databases. We found 9 lncRNAs (NONHSAT157362.1, ENST00000511849.2, lnc-ENPP5-2:7, NONHSAT185560.1, NONHSAT184588.1, lnc-SCN4B-2:2, NONHSAT149915.1, lnc-SGO1-1:1, ENST00000617135.1), 13 mRNAs (BLNK, CLIC5, PRX, SCN4B, ADRA1D, NCKAP5, EGR1, TBX3, EDNRB, ADAMTS8, PTPN21, CD1E, AKR1C2), and 117 TFs were involved in lncRNA-TF-mRNA network (Table S20). Fig. 6A showed that three lncRNAs (NONHSAT157362.1, ENST00000511849.2, and lnc-ENPP5-2:7), five mRNAs (BLNK, CLIC5, PRX, SCN4B, ADRA1D), and 209 TFs form a lncRNA-TF-mRNA network with a degree >2 (Fig. 6A). Furthermore, the lncRNA-TF-mRNA network was found to be enriched in the progesterone metabolic process (as illustrated in Fig. 6B and detailed in Table S21). Additionally, enrichment analysis revealed involvement in KEGG pathways such as adrenergic signaling in cardiomyocytes, calcium signaling, and primary immunodeficiency (as shown in Fig. 6C and detailed in Table S22).

#### 3.5.1 lncRNA-miRNA-mRNA network

**Fig. 7:**
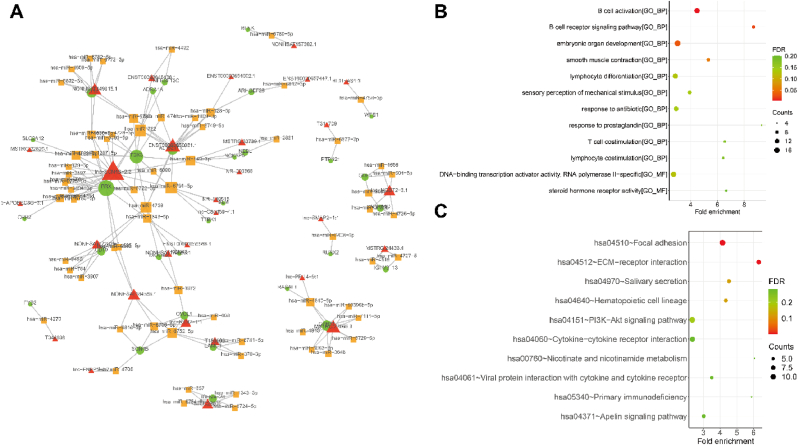
Construction of lncRNA-miRNA-mRNA network **(A).** In the ternary network of the top 100 lncRNA-miRNA-mRNA, red indicates lncRNAs, orange indicates miRNAs, and green indicates mRNAs. **(B).** The bubble plots of GO enrichment, including top 10 BP, top 10 CC, and top 10 MF terms.**(C).** The bubble plots of top 10 KEGG enrichment. (For interpretation of the references to color in this figure legend, the reader is referred to the Web version of this article.)

Traditionally, lncRNA acts as a ceRNA and regulates mRNA expressions by sequestering microRNAs (miRNAs), thereby modulating their activity. According to the 43,557 lncRNA-mRNA pairs and the miRBase database, a total of 8701 lncRNA-miRNA-mRNA pairs were identified, including 197 lncRNAs, 542 miRNAs, and 275 mRNAs (Table S23). As depicted in Fig. 7A, the lncRNA-miRNA-mRNA ceRNA network was established based using the top 100 correlation coefficients between lncRNA-mRNA pairs. The protein-coding transcripts from this ceRNA network were enriched in various biological processes (as demonstrated in Fig. 7B and detailed in Table S24). Furthermore, these protein-coding transcripts were mainly related to pathways like focal adhesion and receptor-ECM interaction (as illustrated in Fig. 7C and detailed in Table S25).

### 3.6 Immune pathway-related LncRNAs analysis

**Fig. 8:**
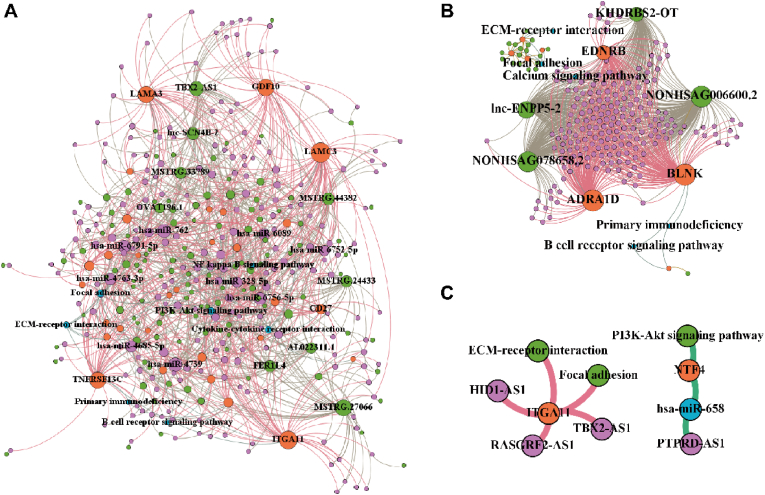
Immune pathway-related lncRNAs analysis **(A).** The network of lncRNA-miRNA-mRNA-immune pathways and *trans*-regulatory lncRNA-mRNA-immune pathways. Green indicates lncRNAs, purple indicates miRNAs, orange indicates protein-coding transcripts, and blue indicates immune-related pathways. **(B).** The network of *trans*-regulatory lncRNA-mRNA-immune pathways. Green indicates lncRNAs, purple indicates miRNAs, orange indicates protein-coding transcripts, and blue indicates immune-related pathways. **(C).** PTPRD-AS1-miR-658-NTF4-PI3K/AKT signaling pathway and *trans*-regulatory lncRNAs (HID1-AS1, RASGRF2-AS1, TBX2-AS1)-ITGA11-immune pathways (focal adhesion, ECM-receptor interaction) were validated based on TCGA database. (For interpretation of the references to color in this figure legend, the reader is referred to the Web version of this article.)

Previous analysis revealed that lncRNAs and the protein-coding transcripts were involved in immune-related pathways. Therefore, we focused on the immune pathway enrichment analysis in this study. GO enrichment analysis discovered that *cis*-regulatory lncRNAs were enriched in the modulation of the B cell receptor signaling. *Trans*-regulatory lncRNAs were found to be enriched in various biological processes related to B cell function (Fig. 8A, Table S26). KEGG pathway enrichment analysis indicated that lncRNAs from ceRNA enriched in receptor-ECM interaction and focal adhesion, and *cis*-regulatory lncRNAs enriched in primary immunodeficiency. (Fig. 8B, Table S27). Furthermore, we validated the ceRNA network and other lncRNA-mRNA pairs, including *cis*-/*trans*-/TF-regulatory lncRNA-mRNA pairs according to the TCGA database. LncRNA PTPRD-AS1-has-miR-658-NTF4-PI3K-AKT signaling pathway was identified from immune-related ceRNA (Table S28). And six *trans*-lncRNA-mRNA pairs, including HID1-AS1-ITGA11-Focal adhesion, HID1-AS1-ITGA11-ECM-receptor interaction, RASGRF2-AS1-ITGA11-Focal adhesion, RASGRF2-AS1-ITGA11-ECM-receptor interaction, TBX2-AS1-ITGA11-Focal adhesion, TBX2-AS1-ITGA11-ECM-receptor interaction were identified from the immune-related other lncRNA-mRNA pairs (Table S29). Taken together, NTF4, PTPRD-AS, ITGA11, HID1-AS1, RASGRF2-AS1, and TBX2-AS1 were identified from microarray data and validated by TCGA data (Fig. 8B–C). These results suggested that differentially expressed NTF4, PTPRD-AS, ITGA11, HID1-AS1, RASGRF2-AS1, and TBX2-AS1 both in RNA-seq and microarray data.

### 3.7 The potential of hub genes for prognosis

**Fig. 9:**
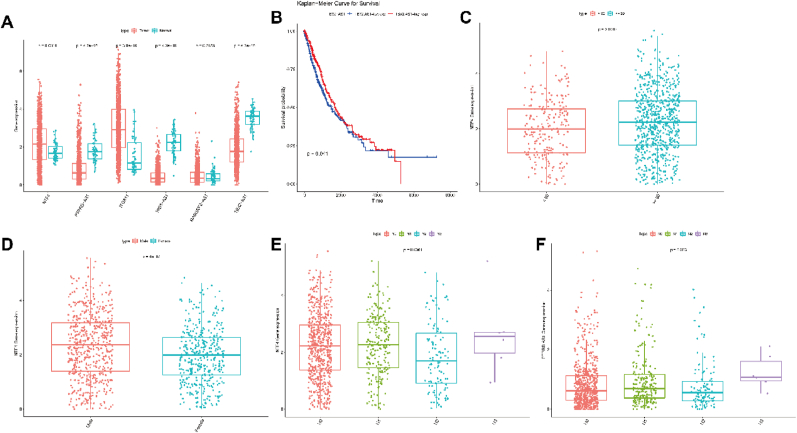
Prognostic potential of ceRNA network and correlation between gene expressions and clinical characteristics **(A).** The box plot revealed the expression of NTF4, PTPRD-AS, ITGA11, HID1-AS1, RASGRF2-AS1, and TBX2-AS1 between NSCLC tumor and normal tissues. **(B).** Overall survival curves of high/low expression of TBX2-AS1 in NSCLC. **(C).** Correlation between the expression of NTF4 and age. **(D).** Correlation between the expression of NTF4 and gender. **(E).** Correlation between the expression of NTF4 and N stage. **(F).** Correlation between the expression of PTPRD-AS and N stage.

**Fig. 10:**
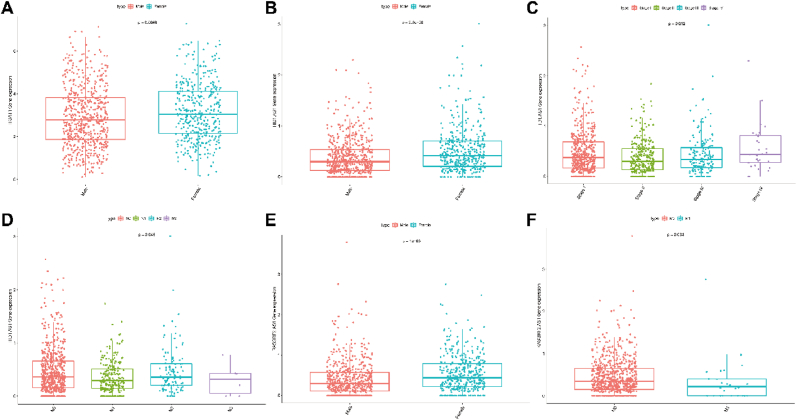
Correlation between gene expressions and clinical characteristics. **(A)–(F).** Correlation between the expression of ITGA11, HID1-AS1, RASGRF2-AS1 and clinical characteristics (age, gender, clinical stages, T/N/M stages).

Finally, we predicted the potential of the ceRNA network for prognosis based on TCGA data. We found RASGRF2-AS1, NTF4, and ITGA11 are upregulated, but PTPRD-AS, HID1-AS1, and TBX2-AS1 are downregulated in NSCLC tumors compared with those in normal tissues (Fig. 9A). High expression of TBX2-AS1 revealed a favorable survival time for NSCLC patients (Fig. 9B), and the survival analyses of the remaining genes were not statistically different (P > 0.05, Figs. S4A–E). Correlation analysis unveiled the following associations: NTF4 expression correlated with older age, gender, and lymph node metastasis (Fig. 9C–E); The correlation between this gene and primary tumour, distal metastasis and clinical analysis was not statistically different (P > 0.05, Figs. S5A–C). PTPRD-AS expression was linked to lymph node metastasis (Fig. 9F); The results of the correlation analysis between this gene and the remaining factors were not statistically different (P > 0.05, Figs. S5D–H). ITGA11 expression showed a relationship with gender (Fig. 10A); HID1-AS1 expression was related to gender, lymph node metastasis, and clinical stages (Fig. 10B–D); RASGRF2-AS1 expression exhibited associations with gender and distant metastasis (Fig. 10E–F). The remaining analyses that were not statistically significant are shown in the pictures (Figs. S6A–L). None of the results of the correlation analyses of TBX2-AS1 were statistically significant (p > 0.05, Figs. S7A–F). Therefore, the expression of RASGRF2-AS1, TBX2-AS1, NTF4, PTPRD-AS, ITGA11, and HID1-AS1 revealed the predicated potential for NSCLC patients.

## 4 Discussion

In recent decades, advancements in high-throughput technologies in genomics and omics led to the identification of numerous coding and non-coding transcripts implicated in tumorigenesis and cancer progression [38,39]. Using bioinformatics, we comprehensively and deeply acknowledged the gene expression and their regulatory mechanism in NSCLC, instead of the traditional gene-by-gene approaches in research [40].

In this study, 391 differentially expressed long non-coding RNAs (lncRNAs) and 344 transcripts were identified in NSCLC tumors in contrast to adjacent normal tissues. Furthermore, these differentially expressed transcripts were found to be enriched in several immune-related signaling pathways. Through co-expression analysis of lncRNAs and mRNAs, we identified 27 *cis*-regulatory lncRNAs, and 34 *trans*-regulatory lncRNAs, and subsequently constructed a ceRNA network. This network comprises 8701 lncRNA-miRNA-mRNA pairs. Moreover, 9 lncRNAs co-expressed TFs were identified from 27 *cis*-regulatory lncRNAs, and the lncRNA-TF-mRNA network was constructed. We also identified that the six transcripts (NTF4, PTPRD-AS, ITGA11, HID1-AS1, RASGRF2-AS1, TBX2-AS1) are associated with immune and the prognosis of NSCLC.

In general, nuclear-localized lncRNAs play cis and *trans*-regulatory roles in regulating gene transcription, mRNA splicing, DNA elements, and nuclear architecture [21,41,42]. Cytoplasmic-localized lncRNAs act as ceRNA roles to impact RNA stability and miRNA activity [43]. Previous studies have highlighted the pivotal roles of lncRNAs in tumorigenesis and tumor progression. For instance, the prognostic value of IPTX1 in breast cancer has been demonstrated through bioinformatics analysis [44]. Eleven hub genes are associated with papillary thyroid carcinoma [45]. Unlike cytoplasmic-localized lncRNAs, which have been widely reported in tumors [46,47], nuclear-localized lncRNAs regulate various complex mechanisms in the tumor. For example, lincRNA-Cox2 not only functions as an enhancer RNA to regulate Ptgs2 but also acts as a *trans*-regulatory lncRNA to regulate Ptg2 in innate immune [48]. And lncRNA RBAT1 promotes tumor initiation by interacting with and activating HNRNPL and E2F3 in bladder cancer, respectively [49]. Besides, lncRNA NEF inhibits hepatocellular carcinoma growth, metastasis, and epithelial-to-mesenchymal transition (EMT) through *cis*-activating FOXA2 and inactivating the Wnt/β-catenin signaling pathway [50]. Rare lncRNAs have been discovered to regulate tumorigenesis and progression either in cis or trans. For example, HMGA1-lnc has been identified as a novel *cis*-regulatory lncRNA that negatively regulates the expression of HMGA1 in NSCLC [51]. lncRNA TBILA accelerates NSCLC progression by activating HGAL expression in cis and lighting S100A7/JAB1 signaling pathway [52]. The above studies have indicated that lncRNAs regulate tumorigenesis and progression in cis and trans are the vital regulatory mechanism in tumors.

Dysregulation of the immune system influences the course of tumour development. Most studies related to immune regulation and immunotherapy have focused on coding genes. An increasing number of articles show that lncRNAs have a crucial function in immunomodulation in the tumour microenvironment [53]. Here, we identified 27 *cis*-regulatory lncRNAs and 34 *trans*-regulatory lncRNAs in NSCLC, which are involved in several immune pathways, including primary immunodeficiency, focal adhesion, and ECM-receptor interaction. It has been found that primary immunodeficiency is a potential risk factor for malignancies [54,55]. It has been proposed that immunodeficiency may affect the efficacy of chemotherapy [56]. In a revealing transcriptomic study of NSCLC, B cells were shown to exist in multiple subtypes, suggesting multiple roles in the NSCLC microenvironment [57]. Our article shows that *cis*-regulatory lncRNAs are predominantly enriched in pathways of primary immunodeficiency and enriched in the regulation of B-cell receptor signalling. This result may provide new research directions for therapeutic approaches targeting immunity. Indeed, focal adhesion signaling plays a crucial role in tumor cell migration and is strongly related to cancer cell resistance to both radiotherapy and chemotherapy [[58], [59], [60]]. A previous study has also demonstrated that CX3CL1 promotes lung cancer cell migration and invasion by activating focal adhesion signaling [60]. Additionally, G9A has been shown to accelerate NSCLC cell metastasis and invasion by enhancing focal adhesion kinase activation [61]. In addition, lots of research has indicated ECM-receptor interaction pathway is involved in multiple tumors [[62], [63], [64], [65]]. We found that *trans*-regulated lncRNA-mRNA pairs are mainly enriched in immune-related biological processes. In a study based on single-cell sequencing data, the authors proposed that lncRNAs are associated with immune cell infiltration, and the article found that lung cancer-associated lncRNAs showed a trend of high expression in immune cell populations (B cells, T cells, etc.) [57]. lncRNA NRON was found to regulate the resting state of T cells [66]. Our findings may contribute a theoretical basis for the role of lncRNAs in immune regulation.

In this study, we integrated and analyzed *cis*-/*trans*-regulatory lncRNAs, and the lncRNAs that function as ceRNA, the prognostic values of the six transcripts (NTF4, PTPRD-AS, ITGA11, HID1-AS1, RASGRF2-AS1, TBX2-AS1) were then validated based on TCGA data. The results revealed that the PTPRD-AS1-NTF4-PI3K/AKT signaling pathway and the three *trans*-regulatory lncRNAs (HID1-AS1, RASGRF-AS1, TBX2-AS1) regulated the ITGA11 in *trans*. The previous study has identified PTPRD-AS1 as a novel lncRNA involved in bladder cancer, with associations with immune-related processes [67,68]. Our findings corroborate that PTPRD-AS1 acts as a tumor suppressor and participates in immune-related pathways. Additionally, NTF4, known as a neurotrophic factor, has been implicated in promoting the growth and metastasis of colorectal cancer and gastric cancer cells [69,70]. The role of NTF4 in NSCLC hasn't been reported yet. The *cis*-regulatory lncRNAs, HID1-AS1, RASGRF2-AS1, and TBX2-AS1, were first identified in NSCLC, of these, HID1-AS1 and TBX2-AS1 acted as the tumor suppressors, whereas RASGRF2-AS1 upregulated in NSCLC. Besides, our data accord with the previous study, ITGA11 has been found to play as an oncogene and upregulated in NSCLC [[71], [72], [73]]. ITGA11 shown to be involved in regulating resistance to tumour therapy drugs in gastric [74] and pancreatic cancers [75]. Immunotherapy has emerged as a key modality in the treatment of malignant tumors in recent years, however, due to limitations, we were not able to explore the relationship between key lncRNAs and immune checkpoints in the current study. KRAS, KEAP1 and STK11 in NSCLC have been shown to play a key role in immunotherapy [76]. In subsequent studies, we may try to find the relationship between key lncRNAs and these genes.

In summary, our analysis based on clinical samples demonstrated the potential of LncRNAs in regulating NSCLC. In subsequent studies, further determination of how key lncRNAs regulate tumour progression through their target genes, e.g., affecting cancer cell migration, invasion, apoptosis, and other actions and mechanisms by animal or cellular models will facilitate the discovery of more lncRNA-based therapeutic approaches. Exploring the drug resistance of lncRNAs in NSCLC will also be one of the future research directions, which may provide research strategies for drug sensitivity during NSCLC treatment. In addition, investigating the correlation of lncRNA with the expression of immune cell markers in relation to immune checkpoints may provide a theoretical basis for the effectiveness of future immunotherapy for NSCLC.

## 5 Conclusion

Our study first discovers the different lncRNAs regulatory mechanisms in NSCLC. We identified the *cis*-, *trans*-, and ceRNA-regulatory mechanisms in NSCLC, and associated with immune-related pathways. Our data provided the important theoretical basis of lncRNAs regulating tumorigenesis and progression in NSCLC.

## CRediT authorship contribution statement

**Yinqiang Liu:** Writing – original draft. **Hongjv Yang:** Writing – review & editing. **Guoli Lv:** Data curation. **Jin Duan:** Formal analysis. **Wei Zhao:** Resources. **Yunfei Shi:** Conceptualization. **Youming Lei:** Conceptualization.

## Data availability statement

All data presented in this study were included.

## Ethics statement

This study involving human specimens was approved by the Ethics Committee of the First Hospital of Kunming Medical University. All participants have known the purposes of this study and gave written informed consent.

## Funding

Yunnan Province Medical Academic Leader Training Object (D-2017013). Applied Basic Research Key Project of Yunnan (2017BS029).

## Declaration of competing interest

The authors declare that they have no known competing financial interests or personal relationships that could have appeared to influence the work reported in this paper.
