# Supplementary material for: Integration analysis of cis- and trans-regulatory long non-coding RNAs associated with immune-related pathways in non-small cell lung cancer
Source: Biochem Biophys Rep. 2024 Oct 28;40:101832. doi: 10.1016/j.bbrep.2024.101832 (PMC11558640; doi:10.1016/j.bbrep.2024.101832)
Supplement: Multimedia component 1 [file mmc1.docx]

**Supplementary Figure 1.** Quality control and data normalization.

**(A).** The box plots exhibited the distribution of raw data and normalized data on samples.

**(B)** and **(E).** The correlation plot illustrated the correlation between the control group (normal) and cases group (tumor) based on lncRNAs and mRNAs data.

**(C)** and **(F).** The PCA plot showed the clustering of lncRNA and mRNA in different samples in two dimensions.

**(D)** and **(G).** The PCA plot t showed the clustering of lncRNA and mRNA in different samples in three dimensions.


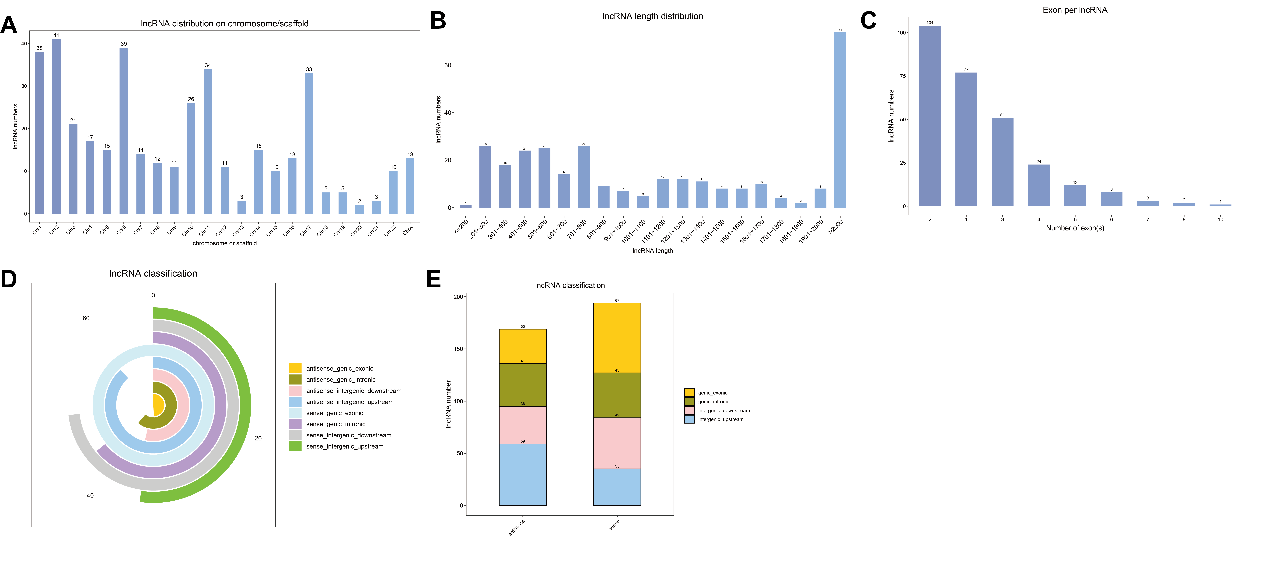


**Supplementary Figure 2**. The features of lncRNAs of NSCLC.

**(A).** The bar plots showed the lncRNA distribution on the chromosome of NSCLC.

**(B).** The bar plots showed the lncRNA length distribution.

**(C).** The bar plots illustrated the number of lncRNAs of each exon.

**(D)-(E).** The circus and bar plots showed the counts and types of different lncRNAs.


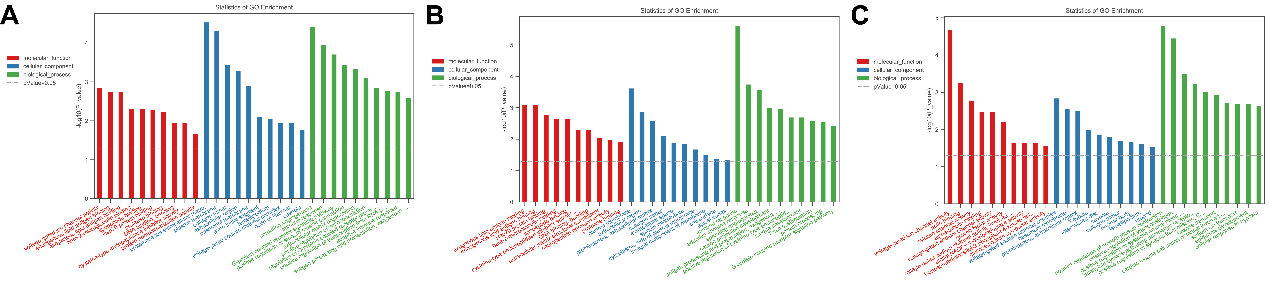


**Supplementary Figure 3**. GO terms enrichment analysis.

**(A).** The bar plots indicated GO enrichment (MF, CC, BP) of all DE-mRNAs.

**(B).** The bar plots indicated GO enrichment (MF, CC, BP) of upregulated DE-mRNAs.

**(C).** The bar plots indicated GO enrichment (MF, CC, BP) of downregulated DE-mRNAs.


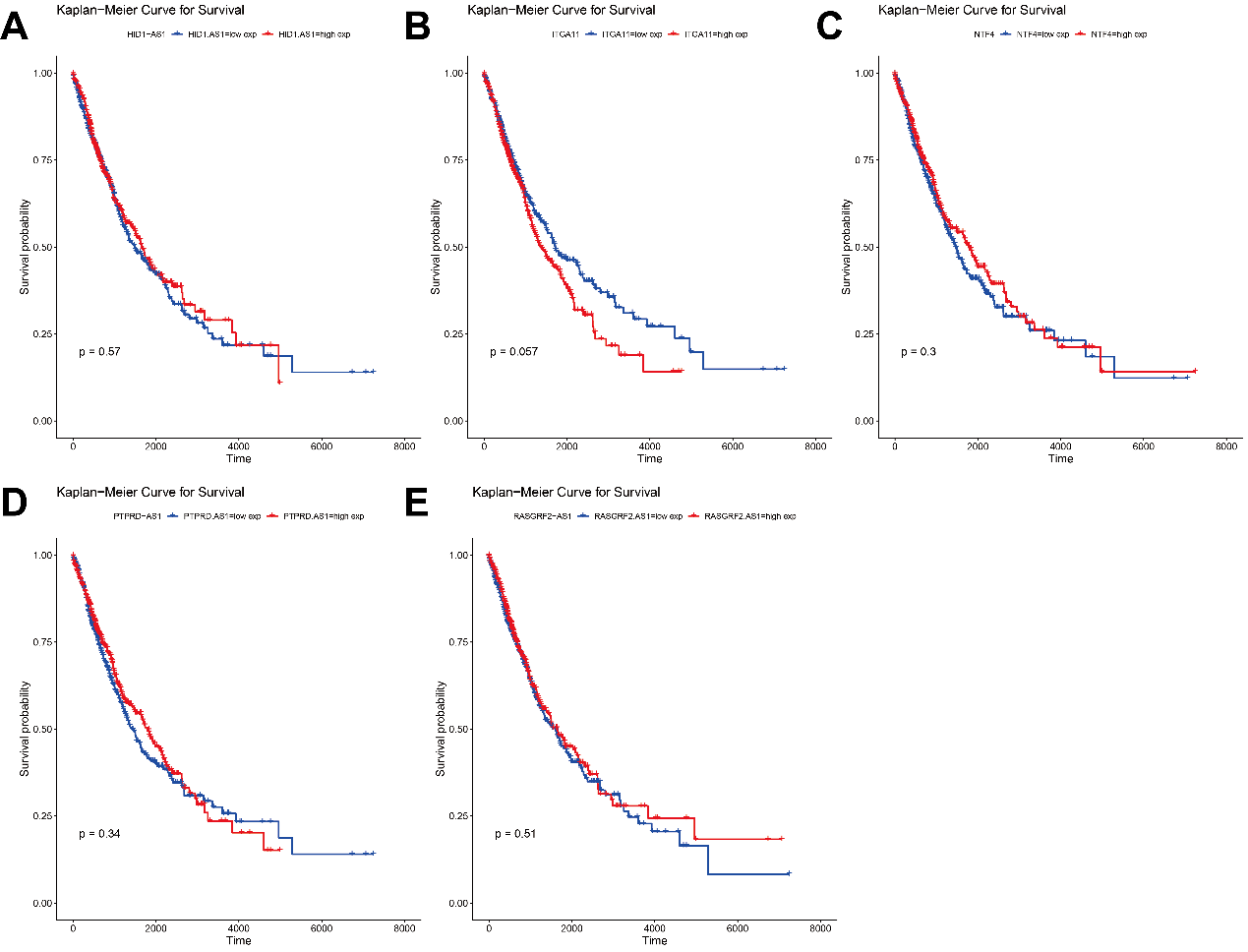


**Supplementary Figure 4.** Overall survival curves

**(A-E).** Overall survival curves of high/low expression of HID1-AS1, ITGA11, NTF4, PTPRD-AS, RASGRF2-AS1 in NSCLC.


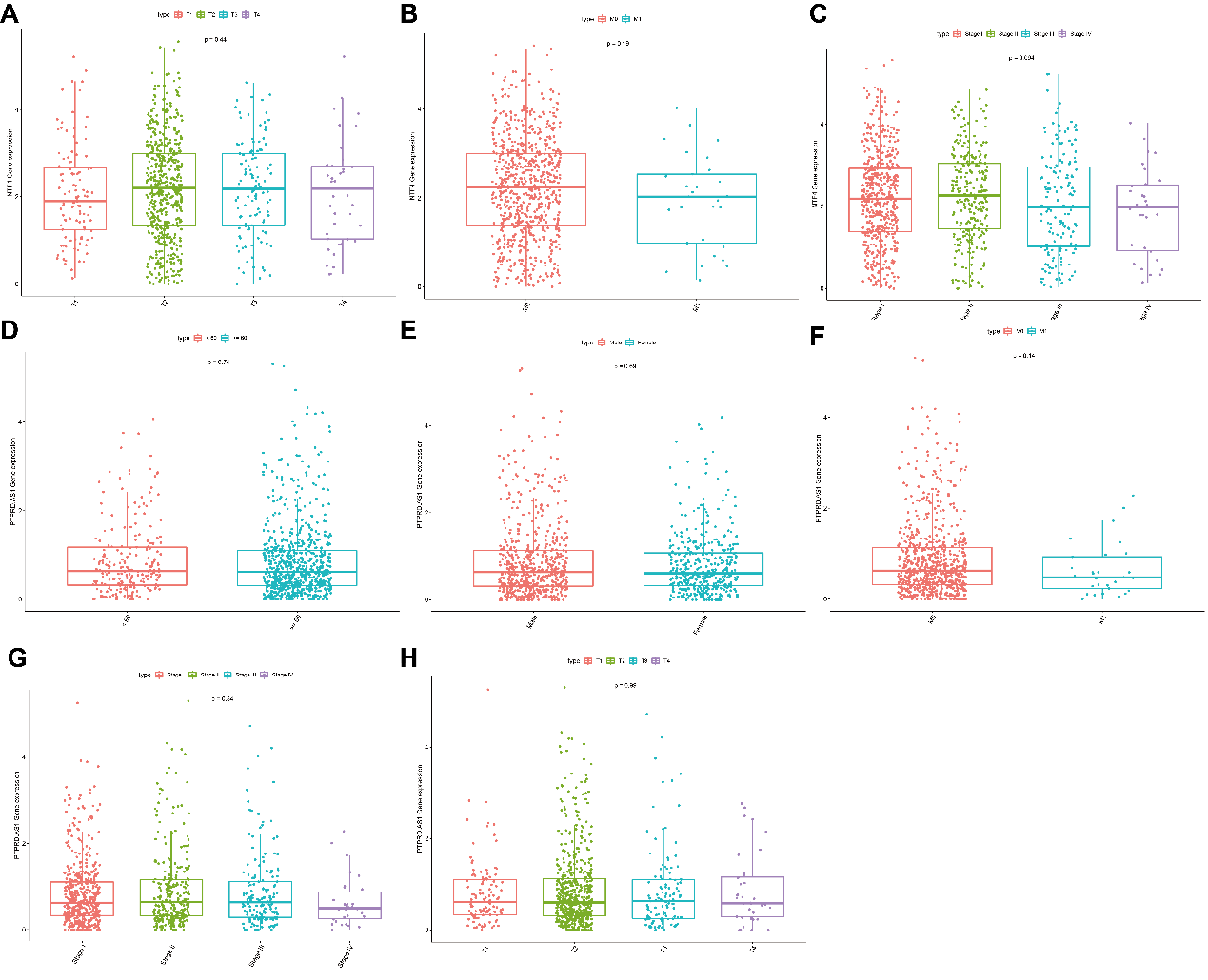


**Supplementary Figure 5.** Correlation between gene expressions and clinical characteristics

**(A-H).** Correlation between the expression of NTF4, PTPRD-AS and clinical characteristics (age, gender, clinical stages, T/N/M stages).


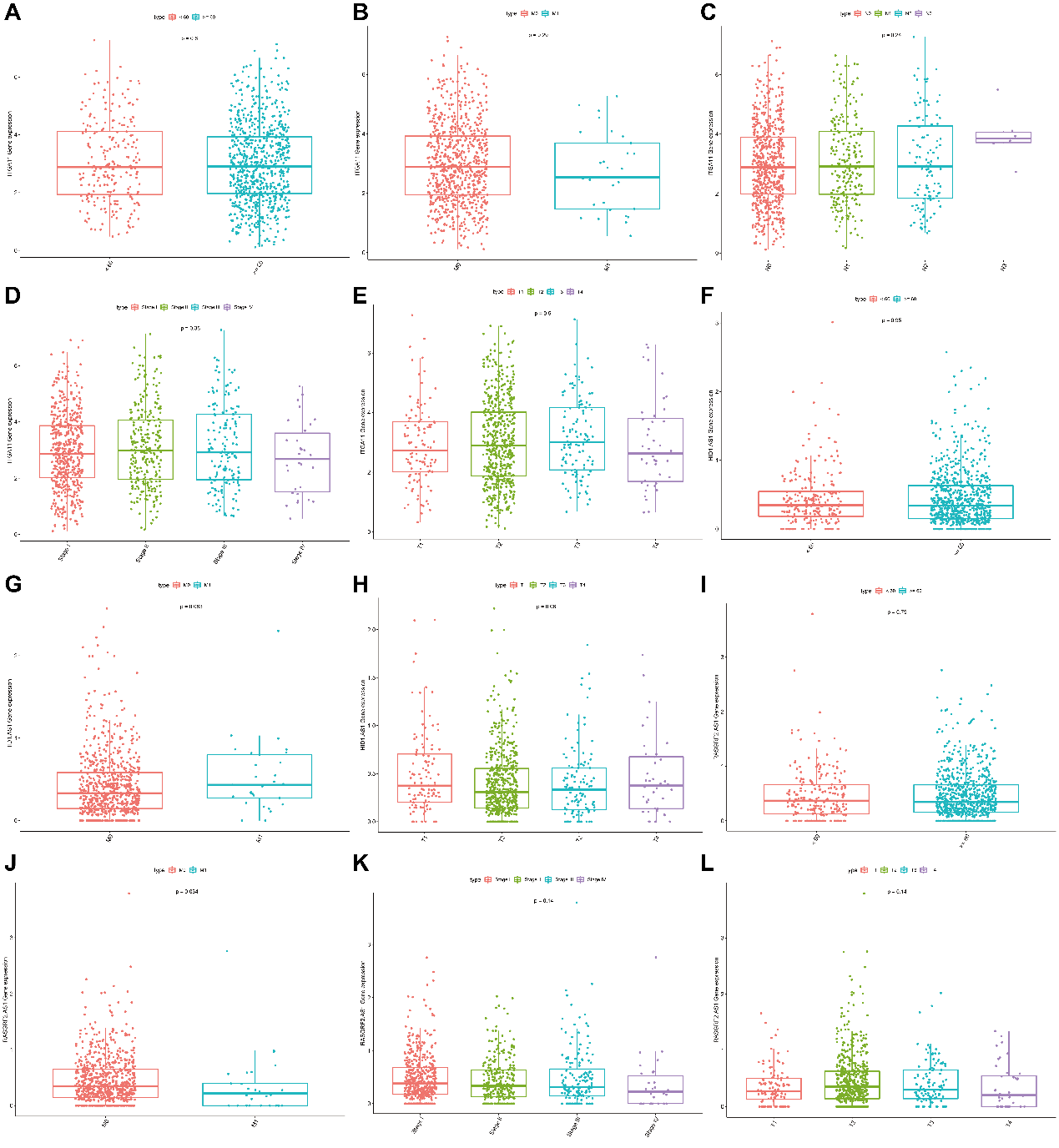


**Supplementary Figure 6.** Correlation between gene expressions and clinical characteristics

**(A-L).** Correlation between the expression of ITGA11, HID1-AS1, RASGRF2-AS1 and clinical characteristics (age, gender, clinical stages, T/N/M stages).


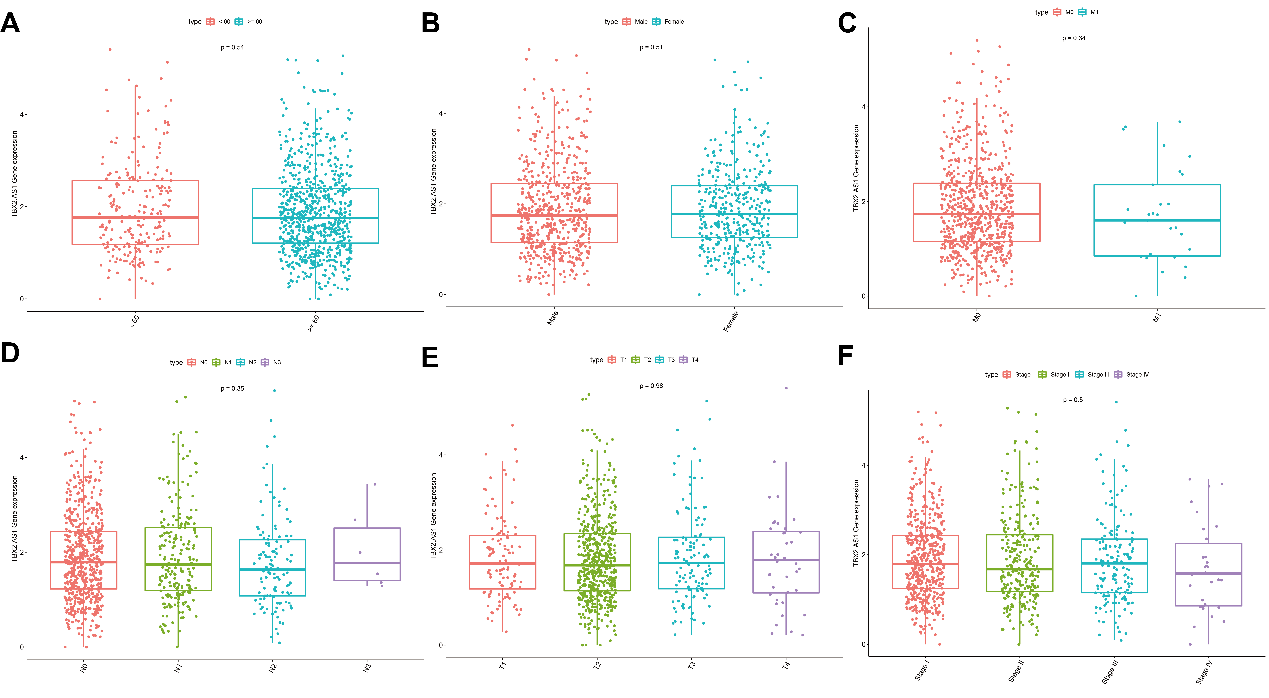


**Supplementary Figure 7.** Correlation between gene expressions and clinical characteristics

**(A-F).** Correlation between the expression of TBX2-AS1 and clinical characteristics (age, gender, clinical stages, T/N/M stages).

**Supplementary Table legend**

Table S1 Differently expressed lncRNA

Table S2 Differently expressed mRNA

Table S3 all_GO_enrichment_result

Table S4 up_GO_enrichment_result

Table S5 down_GO_enrichment_result

Table S6 all_KEGG_enrichment_result

Table S7 up_KEGG_enrichment_result

Table S8 down_KEGG_enrichment_result

Table S9 correxpress_result_lncRNA_mRNA

Table S10 lncRNA_GO_result

Table S11 summary_GO

Table S12 lncRNA_KEGG_result

Table S13 summary_KEGG

Table S14 summary_cis lncRNA-mRNA pairs

Table S15 CISPCs_All enrich GO

Table S16 CISPCs_All enrich KEGG

Table S17 summary_trans

Table S18 transPCs_All enrich GO

Table S19 transPCs_All enrich KEGG

Table S20 lncRNA_TF_mRNA_result (lnc from cis)

Table S21 TFPCs_All enrich GO

Table S22 TFPCs_All enrich KEGG

Table S23 lncRNA_miRNA_mRNA

Table S24 ceRNAPCs_All enrich GO

Table S25 ceRNAPCs_All enrich KEGG

Table S26 immune_GO Term

Table S27 immune_KEGG pathways

Table S28 circRNA_miRNA_mRNA

Table S29 lncRNA_miRNA_mRNA
